# Meteorological factors affect the epidemiology of hemorrhagic fever with renal syndrome via altering the breeding and hantavirus-carrying states of rodents and mites: a 9 years’ longitudinal study

**DOI:** 10.1038/emi.2017.92

**Published:** 2017-11-29

**Authors:** Fachun Jiang, Ling Wang, Shuo Wang, Lin Zhu, Liyan Dong, Zhentang Zhang, Bi Hao, Fan Yang, Wenbin Liu, Yang Deng, Yun Zhang, Yajun Ma, Bei Pan, Yalin Han, Hongyan Ren, Guangwen Cao

**Affiliations:** 1 Department of Acute Infectious Diseases, Municipal Center of Disease Control and Prevention of Qingdao, Qingdao, China; 2 Department of Epidemiology, Second Military Medical University, Shanghai, China; 3 Centre of Disease Control and Prevention of Huangdao District, Qingdao, China; 4 Institute of Epidemiology and Microbiology, Huadong Research Institute for Medicine and Biotechnics, Nanjing, China; 5 Department of Tropical Infectious Diseases, Second Military Medical University, Shanghai, China; 6 Institute of Geographic Sciences and Natural Resources Research, Chinese Academy of Sciences, Beijing, China

**Keywords:** China, hantavirus, hemorrhagic fever with renal syndrome, rodent, meteorological factors

## Abstract

The incidence of hemorrhagic fever with renal syndrome (HFRS) in Qingdao, China was three times higher than that of the average national level. Here we characterized the epidemiology, ecological determinants and pathogen evolution of HFRS in Qingdao during 2007–2015. In this longitudinal study, a total of 1846 HFRS patients and 41 HFRS-related deaths were reported. HFRS in Qingdao peaked once a year in the fourth quarter. We built a time series generalized additive model, and found that meteorological factors in the previous quarter could accurately predict HFRS occurrence. To explore how meteorological factors influenced the epidemic of HFRS, we analyzed the relationship between meteorological factors and hantavirus-carrying states of the hosts (including rodents and shrews). Comprehensive analysis showed humidity was correlated to high host densities in the third quarter and high hantavirus-carrying rates of animal hosts in the third to fourth quarters, which might contribute to HFRS peak in the fourth quarter. We further compared the L segments of hantaviruses from HFRS patients, animal hosts and ectoparasites. Phylogenetic analysis showed that hantaviruses in gamasid and trombiculid mites were the same as those from the hosts. This indicated mites also contributed to the transmission of hantavirus. Furthermore, Hantaan virus from HFRS patients, hosts and mites in Qingdao formed a distinct phylogenetic cluster. A new clade of Seoul virus was also identified in the hosts. Overall, meteorological factors increase HFRS incidence possibly via facilitating hosts’ reproduction and consequent mite-mediated hantavirus transmission. New hantavirus subtypes evolved in Qingdao represent new challenges of fighting against HFRS.

## INTRODUCTION

Hemorrhagic fever with renal syndrome (HFRS) is a rodent-borne disease characterized by fever, back pain, headache, hypotension, multisystemic haemorrhage and acute kidney damage. It is caused by single-stranded RNA viruses of the genus *Hantavirus* in the family *Bunyaviridae*.^1, 2^ Rodents are the reservoirs of human pathogenic hantaviruses. Of various hantaviruses identified worldwide, Hantaan virus (HTNV) mostly carried by *Apodemus agrarius* and Amur virus carried by *Apodemus peninsulae* in East Asia, Puumala virus carried by *Clethrionomys glareolus* and Dobrava-Belgrade virus carried by *Apodemus flavicollis* in Europe, and Seoul virus (SEOV) mostly carried by *Rattus norvegicus* worldwide are the pathogens of HFRS; while other hantaviruses cause hantavirus pulmonary syndrome and related outbreaks in the Americas.^1, 2, 3, 4, 5^

China was seriously affected by HFRS, with 1 557 622 patients and 46 427 related deaths reported during 1950–2007.^6^ HTNV and SEOV are the main pathogens. HTNV frequently causes severe HFRS even death in rural areas; whereas SEOV causes relative mild disease commonly in urban areas. HFRS was restricted to the northeast corner of China before 1950, spread rapidly after the 1970s and distributed nationwide in the 1990s.^6, 7, 8^ After the implementation of prophylactic measures including rodent control and vaccination, the HFRS incidence has dramatically decreased since 2000.^9^ However, the incidence is still high in eastern China where new endemic areas have emerged.

Qingdao is a famous tourist port city in eastern China (35°35′–37°09′N, 119°30′–121°00′E) and belongs to the northern temperate maritime monsoon climate zone. It consists of five rural counties (Jiaonan, Pingdu, Jiaozhou, Jimo and Laixi), three rural-urban fringe districts (Laoshan, Chengyang and Huangdao), and three urban districts (Shinan, Shibei and Licang), distributing over 11 282 km^2^ of land; the total population in 2015 was ~9.0 million including 5.3 million farmers. According to the surveillance data, 14 516 HRFS cases and 700 related deaths have been officially registered in Qingdao since the first case was identified in 1974. However, epidemic characteristics and risk factors of HFRS remain elusive. Here we aimed to characterize the epidemiology and influencing factors of HFRS during 2007–2015 and pathogen evolution in hosts (rodents and shrews) in Qingdao.

## MATERIALS AND METHODS

### Epidemiology of HFRS

From January 2007, all HFRS cases were notified to the Municipal Centre for Disease Control and Prevention via the National Notifiable Disease Reporting System by medical practitioners. HFRS was diagnosed according to the criteria issued by the Ministry of Health, China. Demographic information of permanent residents was obtained from the statistical yearbook. Weekly meteorological information was obtained from Qingdao Meteorological Bureau. Since 2009, inactive HTNV and SEOV mixed vaccines (Tianyuan Biopharmacology, Hangzhou, China) were provided to 16- to 60-year-old residents in the five rural counties on voluntary basis. If accepted, residents were inoculated twice in May or June at 2 weeks interval.

### Surveillance of hosts and ectoparasites for hantavirus infection

In the five rural counties, hosts (rodents and shrews) were captured by mousetraps placed out of the houses and in the houses during 2011–2015. The density of hosts (the ratio of hosts captured to traps placed) and the density of hantavirus-positive hosts (the density multiplying the hantavirus-positive rate of hosts) were evaluated every 3 months. Fresh lung tissues of the hosts were tested for *Hantavirus* antigen with monoclonal antibody by direct immunofluorescence assay as previously reported.^10^ We caught ectoparasites from hosts in January and December of 2016 in Jiaonan. The ectoparasites resided in pelage and ears of hosts were immersed in RNA later (Thermo Fisher, Waltham, MA, USA) and then examined using a stereoscopic microscope (Olympus, Tokyo, Japan). The same species from a host were pooled together to extract genomic RNA for the identification of hantaviruses.

### Identification of hantaviruses from patients, hosts and parasites

Viral RNA was extracted from patients with HFRS, the supernatants of hosts’ lung tissues positive for *Hantavirus* antigen, and ectoparasites of each species using Trizol reagent (Invitrogen, Carlsbad, CA, USA). The PrimerScript RT reagent kit (Takara, Dalian, China) was used to generate cDNA from each sample. The L segment (nt.2911-nt.3340) of hantavirus genome was amplified as previously described.^11^ PCR amplicons were sequenced in both directions. The sequences were deposited in GenBank with accession numbers KX775443-KX775467 and KY468937-KY468957. Highly similar control sequences to the L segments of hantaviruses isolated in these hosts during 2011–2015 were automatically searched on BLAST (http://www.ncbi.nlm.nih.gov/BLAST/Blast.cgi). MEGA 5.05 software (download from http://www.megasoftware.net) was employed to align and blunt the control sequences and the sequences from the HFRS patients, hosts and mites. Nucleotide sequences were aligned with the above similar sequence, using Clustal W program implemented in MEGA 5.05. A phylogenetic tree was constructed by the neighbor-joining method (bootstrap: 1000 replicates) as previously described.^12^

### Statistical analysis

In order to explore meteorological effects on the incidence of HFRS, we applied a generalized additive model (GAM). The GAM was formulated as


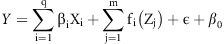


*ε* was random-error term. *Y* was the number of HFRS cases. The effects of meteorological factors on the epidemic of HFRS commonly lagged with certain months including latent period and time for disease transmission. Thus, we conducted a cross correlation analysis to determine the lag periods. We specified the degree of freedom based on the result of cross validation. Year and month as well as monthly temperature, relative humidity, rainfall and sunshine time with a determined lag periodwere initially included in the GAM. Month and sunshine time were excluded during the subsequent selection process. The final GAM in our study was shown as follows:


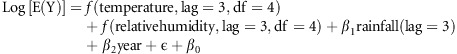


Temperature and relative humidity were included as non-linear function, with a lag time of 3 months, while year, rainfall lagged for 3 months were included as linear functions. Degrees of freedom (df) for both of the non-linear functions were set as four. We divided the data into two parts, one (from 2007 to 2012) served as training set to build the model, the other (from 2013 to 2015) served as validation set to test the model. All data were analyzed by SAS 9.4 (SAS, NC) software. Geographic maps were performed using ArcGIS 9.0 software (ESRI, Redlands, CA, USA).

## RESULTS

### Epidemiological characteristics of HFRS

**Figure 1:**
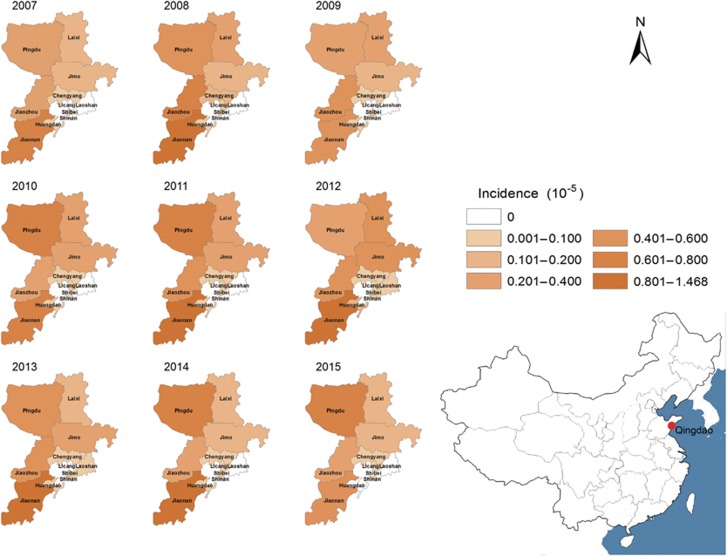
Yearly distribution of HFRS in all districts of Qingdao, China, 2007–2015. Hemorrhagic fever with renal syndrome, HFRS.

A total of 1846 HFRS patients and 41 HFRS-related deaths were reported in Qingdao during 2007–2015. The average age of patients was 46.5 years old (range: 9–91 years). Of those, 1378 (74.7%) were male and 1539 (83.4%) were farmers. The incidence of HFRS varied from 1.64/10^5^ to 3.54/10^5^ each year, with an annual average incidence of 2.45/10^5^. Of the 1846 cases, 1728 (incidence: 3.81/10^5^) were reported in rural counties, 84 (incidence: 0.69/10^5^) in rural-urban fringe zones, and 34 (incidence: 0.19/10^5^) in urban districts (*P*<0.001) (Table 1). Of the 41 fatal cases, 32 (78.1%) were male, 25 (61.0%) were among 41–60 years, 33 (80.5%) were farmers, and 31 (75.6%) died in the third to fourth quarter. The mortality rate was higher in the rural counties than in other districts (*P*<0.001). Jiaonan, Jiaozhou, and Pingdu are the major epidemic areas (Figure 1), accounting for 76.0% cases and 61.0% deaths. The cumulative rate of hantavirus vaccination in the rural counties ranged from 3.68% in 2009 to 12.13% in 2015; the annual incidences of HFRS in the 7 years were not significantly affected by the vaccination (Cochran-Armitage trend test, z=0.670, *P*=0.503) (Table 2).

### Correlation of meteorological factors with the incidences of HFRS

**Figure 2:**
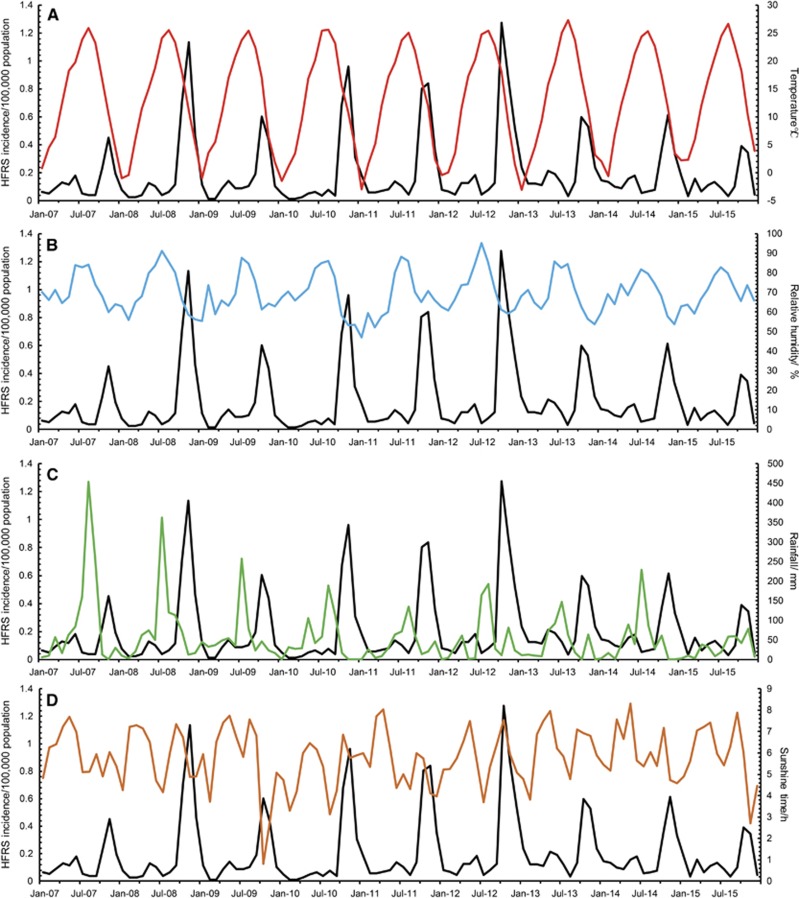
Monthly incidence of HFRS and meteorological information in Qingdao, 2007–2015. The meteorological information from top to bottom are: (**A**) temperature (red); (**B**) relative humidity (blue); (**C**) rainfall (green); (**D**) daily sunshine time (brown), respectively. All black lines represent the monthly number of new cases with HFRS.

**Figure 3:**
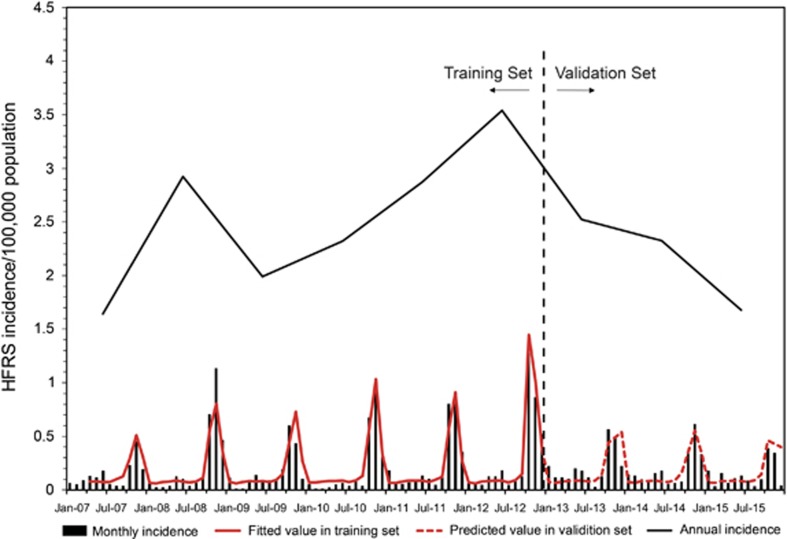
The influences of meteorological factors on the incidence of HFRS in Qingdao, China, 2007–2015. A generalized additive model (GAM) was adopted to fit the HFRS incidence with meteorological factors. Data were divided into two parts. The first 6 years served as training set to build the GAM and the last 3 years were taken as validation set. After selection, temperature, relative humidity and precipitation in the previous quarter were included in the model. Hemorrhagic fever with renal syndrome, HFRS.

**Figure 4:**
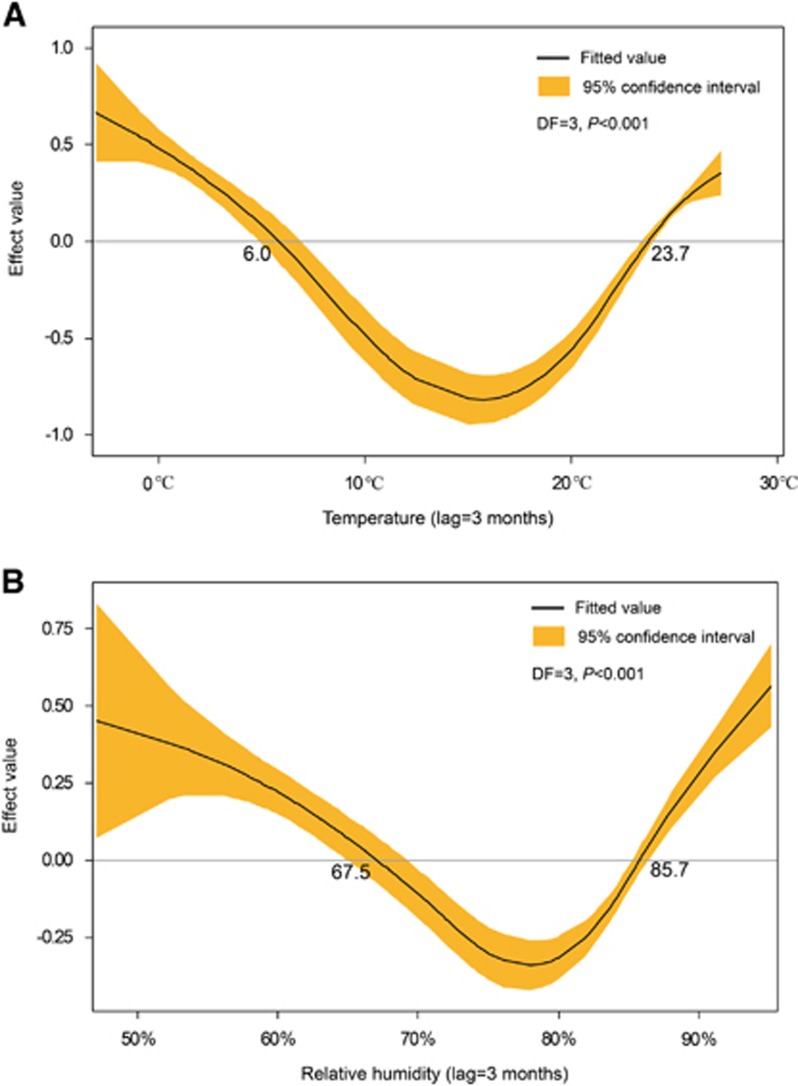
The effect values of temperature and relative humidity on the epidemic of HFRS. (**A**) The effects of temperature with 3 months lag on the incidence of HFRS. (**B**) The effects of relative humidity with 3 months lag on the incidence of HFRS. The effect value above 0 means a positive effect, while below 0 means a negative effect; degree of freedom, DF; hemorrhagic fever with renal syndrome, HFRS.

With a lag time of a quarter, temperature, relative humidity, and precipitation showed the maximum correlation to the incidences of HFRS (Figure 2). After selection, meteorological factors (temperature, humidity, and precipitation) in the previous quarter and year were included in the GAM. Among them, temperature and relative humidity fitted the natural cubic spline equation, while other variables were included as linear equations. The predicted value obtained from the GAM fitted the actual value well during 2013–2015, with an overall determination coefficient of 91.2% (Figure 3). In the linear part of the GAM, precipitation lagged for 3 months (*β*=0.003, *P*<0.001) and year (*β*=0.03, *P*<0.001) were positive with the HFRS incidence. In the non-linear part of the GAM, temperature above 23.7 °C and below 6 °C showed positive effects on the HFRS incidence of 3 months later, whereas temperature between 6.0 °C to 23.7 °C was negative with the HFRS incidence. Similar trends were also observed for relative humidity. Relative humidity above 85.7% and below 67.5% was positive with the HFRS incidence of 3 months later, while negative effect was observed in the rest range (Figure 4). Due to the limited value under 5 °C, the 95% CI of effect value was wider than that in the other temperature range. The same was true for relative humidity. The 95% CI of effect value of relative humidity under 65% was wider than that of other relative humidity ranges. Extreme temperature or humidity showed positive effect on the HFRS incidence, indicating extreme weather might promote the epidemic of HFRS.

### Species, density, and hantavirus-carrying states of hosts

A total of 4081 rodents and shrews were captured using 111 154 valid snap-traps set in the rural counties during 2011–2015. Types of captured rodents were *Apodemus agrarius*, *Rattus norvegicus*, *Mus musculus*, *Rattus rattus*, *Cricetulus triton*, and *Cricetulus arabensis*. Also, *Sorex araneus,* a kind of shrew, was also captured. Of those hosts, 2116 were trapped out of the houses and 1965 in the houses. Among hosts trapped out of the houses, *Apodemus agrarius*, *Rattus norvegicus*, and *Cricetulus triton* accounted for 73.44% of those trapped in the houses, *Rattus norvegicus* and *Mus musculus* accounted for 88.14%. Direct immunofluorescence assay showed that *Apodemus agrarius*, *Rattus norvegicus*, and *Mus musculus* were positive for hantavirus, with a positive rate of 4.37%, 3.56% and 2.14%, respectively (Table 3). The density and hantavirus-positive rate of *Apodemus agrarius* were higher in the third to fourth quarters than in the first to second quarters (0.89% vs 0.26%, *P*<0.001; 4.53% vs 3.80%, *P*=0.002). Overall density and the density of hantavirus-positive *Apodemus agrarius* were significantly higher in the third quarter than in the other quarters (1.12% vs 0.42%, *P*<0.001; 0.05% vs 0.02%, *P*=0.005). The density and hantavirus-carrying rates of *Mus musculus* and *Rattus norvegicus* were not significantly different among quarters. Therefore, the density and hantavirus-positive rate of *Apodemus agrarius* contribute to an increased incidence of HFRS in the fourth quarter.

### Correlation of HFRS incidences, the densities of hantavirus-positive hosts, and meteorological factors in rural counties during 2011–2015

The cumulative incidence of HFRS was significantly higher in Jiaonan than in the other four counties during 2011–2015 (48.5/10^5^ vs 15.3/10^5^, *P*<0.001). This condition was correlated to the densities (5.3% vs 2.1%, *P*<0.001) and the hantavirus-positive rates (3.26% vs 0.42%, *P*<0.001) of hosts, respectively. The quarterly incidence of HFRS was correlated to the hantavirus-positive rates of all hosts (*r*=0.50, *P*=0.028), the density of hantavirus-positive hosts (*r*=0.51, *P*=0.024), the density and hantavirus-positive rate of *Apodemus agrarius* (*r*=0.49, *P*=0.033; *r*=0.64, *P*=0.003), and the densities of hantavirus-positive *Apodemus agrarius* and *Mus musculus* (*r*=0.67, *P*=0.002; *r*=0.64, *P*=0.003) in the previous quarter.

The HFRS incidence was higher in 2012 than in 2011, 2013, 2014, and 2015 (Figure 3). The density of hantavirus-positive hosts was significantly higher in 2012 than in the other 4 years (0.15% vs 0.07% *P*=0.001). Annual average precipitation was higher in 2012 (601.6 mm) than in the other 4 years (485.4 mm on average); the same was true for annual relative humidity (71.4% vs 68.9%).

The HFRS incidence in the fourth quarter was higher in 2012 than in the other 4 years (2.66/10^5^ vs 1.36/10^5^, *P*<0.001). However, the incidences in remaining quarters were not different between 2012 and the other 4 years. The density of hosts in the third quarter was significantly higher in 2012 than in the other 4 years (9.00% vs 3.34%, *P*<0.001). The hantavirus-positive rates of *Apodemus agrarius* were 5.74% and 3.16% in the third quarter and 8.33% and 2.88% in the fourth quarter of 2012 and the other 4 years, respectively. The hantavirus-positive rate of *Apodemus agrarius* and *Mus musculus* in the third and fourth quarters was significantly higher in 2012 than in the other 4 years (5.32% vs 2.67%, *P*=0.032). The density of hantavirus-positive hosts in the third quarter was significantly higher in 2012 than in the other 4 years (0.27% vs 0.06%, *P*<0.001). However, the densities of hosts in the fourth quarter were not different between 2012 and the other 4 years. Average temperature in the third quarter was not different between 2012 (24.36 °C) and the other 4 years (24.37 °C). However, average precipitation in the third quarter was higher in 2012 (383.9 mm) than in the other 4 years (245.0 mm); the same was true for average relative humidity in the third quarter (84.3% vs 79.6%).

### Identification of hantaviruses in ectoparasites of hosts

In January 2016, SEOV (KY468947) was identified from gamasid mites in one of six *Mus musculus* randomly caught in a HFRS-affected village. Interestingly, the rodent was negative for hantavirus. We then set up a field study surrounding croplands of distinct HFRS-affected villages in Jiaonan in December 2016. A total of 80 rodents (47 *Apodemus agrarius*, 18 *Rattus norvegicus*, 15 *Mus musculus*) and seven *Sorex araneus* were caught. Of the 87 hosts, 14 were positive for hantavirus. Surprisingly, two of seven *Sorex araneus* were positive for SEOV (KY468945, KY468940). Ectoparasites mostly escaped overnight from 55 hosts killed by the traps. We then examined the ectoparasites in the remaining 32 hosts (27 *Apodemus agrarius*, two *Mus musculus*, one *Rattus norvegicus*, and two *Sorex araneus*) who were individually packaged minutes after being killed by the traps and transported on ice. A total of 160 gamasid mites (larva and adults on bodies), 371 chigger mites (larva in ears), 16 lice (on bodies) and 1 flea (on body) were separated from the 32 hosts. Interestingly, SEOV (KY468946) was identified in gamasid mites from a hantavirus-negative *Rattus norvegicus*; HTNV (KY468949) was identified in chigger mites from an *Apodemus agrarius* positive for HTNV (KY468950).

### Novel subpopulations of HTNV and SEOV endemic in Qingdao

**Figure 5:**
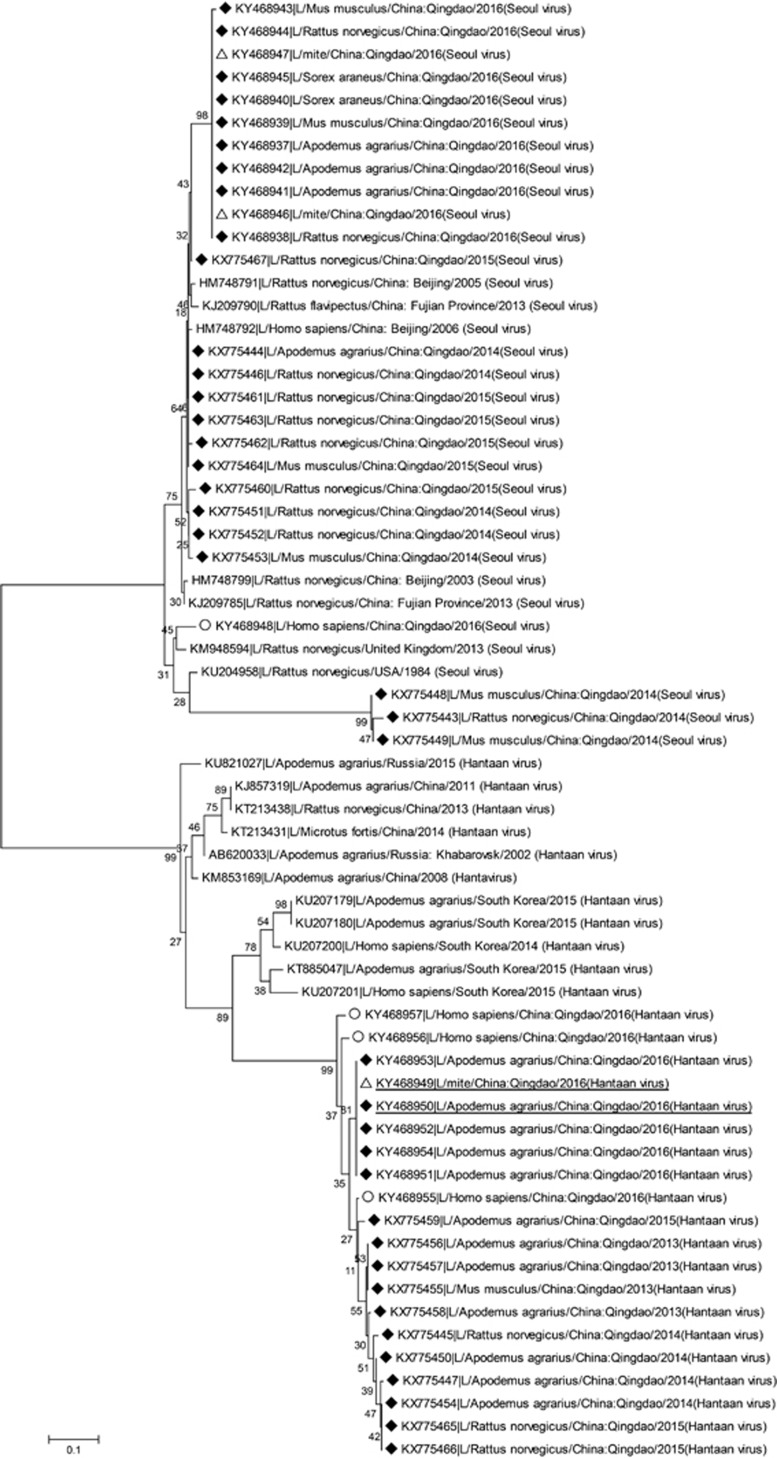
Phylogenetic analyses of 46 hantaviruses (L segment) isolated in Qingdao, China. ♦ Black diamond symbol: 39 isolates from hosts (including rodents and shrews); △White triangle symbol: three isolates from mites; ○White circular symbol: four isolates from HFRS patients. hemorrhagic fever with renal syndrome, HFRS.

Figure 5 showed phylogenetic relationship of hantaviruses isolated in HFRS patients, hosts and mites. HTNV was mostly identified in *Apodemus agrarius* but occasionally in *Rattus norvegicus* and *Mus musculus*, while SEOV was mostly identified in *Rattus norvegicus* and also in *Apodemus agrarius, Mus musculus,* and *Sorex araneus*. The SEOV strains from gamasid mites were the same as those from hosts caught in 2016; HTNV strain from chigger mites was the same as that from *Apodemus agrarius* in 2016. SEOV identified in patients had a little difference in the sequence from the one identified in hosts and mites; the same was true for HTNV. Importantly, HTNV from patients, hosts, and mites in Qingdao formed a distinct cluster; SEOV identified in *Mus musculus* and *Rattus norvegicus* also represented a new clade.

## DISCUSSION

HFRS occurred periodically in Qingdao, peaking in 1986, 1999 and 2012. Here, we found that the annual average incidence was 2.45/10^5^ during 2007–2015, with a peak of 3.54/10^5^ in 2012, while this incidence was 0.83/10^5^ in China during 2006–2012.^9^ HFRS peaks once a year in the fourth quarter in Qingdao but twice a year in Zibo (36°47′N118°3′E),^13^ an inland city located at the northwest of Qingdao with a straight-line distance of 283 km. The dual peaks of HFRS each year in Zibo was proved to be associated with the existence of HTNV and SEOV.^13^ Although HNTV and SEOV were observed, HFRS peaked once a year in Qingdao. The difference between two cities was possibly because Zibo had a temperate continental monsoon climate.^14^ In winter, it is colder and dryer in Zibo than in Qingdao. As temperature below 6.0 °C and relative humidity below 67.5% are positive with the HFRS incidence of 3 months later (Figure 4). These meteorological factors could contribute to the difference in the peaks of HFRS epidemic between Zibo and Qingdao. Thus, understanding how meteorological factors affect HFRS in this port city is of epidemiological importance.

Meteorological factors affect HFRS apparently.^15^ We generated a model including three meteorological factors in the previous quarter that seemed more reliable and practicable in predicting HFRS in the endemic areas than did the previously reported models.^16, 17, 18, 19^ Nevertheless, it is important to know how meteorological factors affect the occurrence of HFRS. Here, we showed that precipitation and relative humidity were positively correlated to the densities of hosts and/or hantavirus-positive hosts in the third quarter and that there was a close temporal association between the densities of hosts and/or hantavirus-positive hosts in the third quarter and the incidence of HFRS in the fourth quarter. The densities of hosts or hantavirus-positive hosts were positively correlated to the incidence of HFRS. The data were quite consistent with previous reports.^10, 17, 18^ Hantavirus transmission among hosts is speculated to be likely maintained through biting during aggressive interaction.^20^ However, it is hard to explain that the hantavirus-positive rates of *Apodemus agrarius* and *Mus musculus* are higher in the fourth quarter on condition that rodent breeding is inactive in this season. Here, we provided direct evidence indicating that hosts’ mites, whose densities could be influenced by humidity, might be important in mediating host-to-host and possibly host-to-human transmission of hantaviruses.

The general belief that hantavirus transmission to humans occurs via inhalation of aerosolized hosts (rodents and shrews) excreta is based on early observations that humans acquired the infection from laboratory rats.^21, 22, 23^ Previous studies in China showed that HTNV could be isolated from gamasid and trombiculid mites collected from the nests of field hosts and from laboratory-reared offspring of these mites and that both trombiculid and gamasid mites could transmit HTNV by biting susceptible mammals.^24, 25^ Furthermore, hantavirus-specific RNA was ever identified from wild trombiculid mites in USA.^26^ In the present study, two SEOV and one HTNV strains were identified in gamasid and trombiculid mites from 38 hosts. The hantavirus-positive rate in the mites should be underestimated because these ectoparasites escaped quickly after their hosts were killed by the traps. Interestingly, two gamasid mite-derived SEOV strains were isolated from hantavirus-negative hosts, indicating the importance of gamasid mites in transmitting hantaviruses among hosts. Gamasid mites live in their hosts’ nests and attach to the host during feeding; the larval trombiculid mites feed on vertebrates. The peak months of trombiculid and gamsid mites on hosts are October and November.^24, 27, 28^ Humidity environment facilitates the survival or breeding of mites.^29, 30^ We believe that humidity facilitates the regeneration and infestation of the mites, thus contributing to the increased hantavirus-positive rates of hosts in the third to fourth quarters in 2012. Thus, humid environment facilitates the regeneration of hosts and mites, the mites mediate host-to-host and possible host-to-human transmission of hantaviruses via biting.

Although the L segment is relatively conservative compared to the S and M sequences, it can represent the phylogenetic relationship of different hantaviruses.^11, 31^ Our phylogenetic analysis using the L segment indicated that HTNV and SEOV evolved each year. Importantly, HTNV from HFRS patients, hosts, and mites in Qingdao formed a distinct new cluster, representing a distinct lineage selected by local ecologic system. The new HTNV strain endemic in Qingdao might be one of the reasons that vaccination against hantaviruses did not significantly reduce the local incidence of HFRS. We also found a new SEOV strain circulated in *Mus musculus Rattus* and *norvegicus*. Clinical significance of this new SEOV strain remains to be determined.

There are several limitations in our study. Our demonstration about the effects of meteorological factors on the epidemic of HFRS is based on ecological study, and this practice might involve confounding factors. Besides the effect of meteorological factors, hosts and mites, there are other factors which could influence the incidence of HFRS, for instance, the increasing exposure to HNTV for the farmers during the third to fourth quarters due to the behaviors of harvest and storage of crops. Though SEOV and HTNV were identified in mites, more investigations were needed to confirm the role of mites in the transmission process. We proved that mites are the potential vectors of pathogen transmission, but we are not sure whether other transmission processes are also involved. Vaccination during 2009–2015 did not significantly reduce the incidences of HFRS, possibly because the vaccination coverage was too low to build immune barriers or altered viral antigenicity due to viral evolution.

Conclusively, HFRS was endemic in rural counties of Qingdao and peaked once a year in the fourth quarter. *Apodemus agrarius*, *Mus musculus, Rattus norvegicus* and *Sorex araneus* were the natural hosts in Qingdao. We developed a GAM model including meteorological factors in the previous quarter and it accurately predicted the epidemic of HFRS in the endemic areas. Precipitation and relative humidity in the third quarter are correlated to the density of hosts in the third quarter and the hantavirus-positive rates of *Apodemus agrarius* and *Mus musculus* in the third to fourth quarters, contributing to the HFRS peak in the fourth quarter. Gamasid and trombiculid mites contribute to hantavirus transmission among hosts and possibly from hosts to humans. HTNV from HFRS patients, hosts and mites in Qingdao formed a distinct new cluster. A new clade of SEOV was identified in *Mus musculus* and *Rattus norvegicus* in Qingdao. New clades of HTNV and SEOV might evolve via adapting to the local ecologic system, representing coming challenges of fighting against HFRS.

## Figures and Tables

**Table 1:** Characteristics of hemorrhagic fever with renal syndrome, Qingdao, China, 2007–2015

**Years**	**Urban districts**		**Rural-urban fringe zones**		**Rural counties**		**Statistical tests**	
	**No. of cases (incidence /10^5^)**	**No. of deaths (mortality /10^5^)**	**No. of cases (incidence/10^5^)**	**No. of deaths (mortality/10^5^)**	**No. of cases (incidence/10^5^)**	**No. of deaths (mortality/10^5^)**	**Incidence^a^**	**Mortality^b^**
2007	1 (0.06)	0 (0)	7 (0.69)	0 (0)	119 (2.42)	4 (0.08)	*χ*^2^=51.5, *P*<0.001	*P*=0.759
2008	2 (0.11)	0 (0)	11 (1.09)	1 (0.10)	214 (4.32)	3 (0.06)	*χ*^2^=94.9, *P* <0.001	*P*=0.314
2009	2 (0.11)	0 (0)	9 (0.89)	1 (0.10)	144 (2.91)	5 (0.10)	*χ*^2^=60.0, *P* <0.001	*P*=0.459
2010	3 (0.16)	0 (0)	4 (0.40)	0 (0)	174 (3.51)	3 (0.06)	*χ*^2^=83.5, *P* <0.001	*P*=0.714
2011	4 (0.19)	0 (0)	10 (0.61)	0 (0)	236 (4.72)	12 (0.24)	*χ*^2^=141.0, *P* <0.001	*P*=0.010
2012	6 (0.29)	0 (0)	21 (1.29)	0 (0)	284 (5.60)	3 (0.06)	*χ*^2^=146.8, *P* <0.001	*P*=0.763
2013	4 (0.19)	0 (0)	11 (0.67)	0 (0)	221 (4.30)	3 (0.06)	*χ*^2^=124.9, *P* <0.001	*P*=0.762
2014	8 (0.38)	0 (0)	9 (0.54)	0 (0)	191 (3.69)	4 (0.08)	*χ*^2^=98.7, *P* <0.001	*P*=0.385
2015	4 (0.19)	0 (0)	2 (0.12)	0 (0)	145 (2.78)	2 (0.04)	*χ*^2^=90.3, *P* <0.001	*P*=1.000
Total	34 (0.19)	0 (0)	84 (0.69)	2 (0.02)	1728 (3.81)	39 (0.09)	*χ*^2^=872.1, *P* <0.001	*χ*^2^=21.3, *P* <0.001

a Chi-square tests was applied to evaluate the difference of HFRS incidence among urban districts, rural-urban fringe zones, and rural countries.

b Fisher exact probability test was applied to evaluate the difference of HFRS mortality among urban districts, rural-urban fringe zones, and rural countries.

**Table 2:** Association of hantavirus vaccination and the incidence of HFRS in rural counties (Jiaonan, Jiaozhou, Pingdu, Jimo, and Laixi) of Qingdao during 2009–2015

**Year**	**2009**	**2010**	**2011**	**2012**	**2013**	**2014**	**2015**
New HFRS cases	144	174	236	284	221	191	145
Accumulative vaccinated population	181 858	316 922	388 584	472 693	473 893	588 449	631 840
Average population	4 940 280	4 952 672	4 996 299	5 072 306	5 135 711	5 177 817	5 208 265
Incidence (/10^5^)^a^	2.91	3.51	4.72	5.60	4.30	3.69	2.78
Accumulative vaccination rate (%)^a^	3.68	6.40	7.78	9.32	9.23	11.36	12.13

a Cochran-Armitage trend test was employed to assess the association of accumulative vaccination rate and the incidence of HFRS, No significant trend was observed (*P*=0.503).

**Table 3:** Prevalence of antibodies against hantaviruses in lung tissues of hosts (including rodents and shrews) by species, Qingdao, China, 2011–2015

**Species**	**No. of hosts captured (density, %) /No. of hosts positive for hantavirus (hantavirus-positive rate, %)^a^**					
	**2011 (16 707 traps)**	**2012 (22 654 traps)**	**2013 (36 166 traps)**	**2014 (19 611 traps)**	**2015 (16 016 traps)**	**Total (111 154 traps)**
*Apodemus agrarius*	9 (0.05)/0 (0)	200 (0.88)/13 (6.50)	303 (0.84)/10 (3.30)	79 (0.49)/4 (5.06)	72 (0.45)/2 (2.78)	663 (0.60)/29 (4.37)
*Rattus norvegicus*	186 (1.11)/1 (0.54)	391 (1.73)/12 (3.10)	352 (0.97)/19 (5.40)	184 (1.15)/ 4 (2.17)	123 (0.77)/8 (6.50)	1236 (1.11)/44 (3.56)
*Mus musculus*	123 (0.74)/0 (0)	307 (1.36)/10 (3.26)	432 (1.19)/10 (2.31)	195 (1.22)/5 (2.56)	157 (0.98)/1 (0.64)	1214 (1.09)/26 (2.14)
*Rattus rattus*	71 (0.42)/0 (0)	4 (0.02)/0 (0)	0 (0)/0 (-)	26 (0.16)/0 (0)	0 (0)/0 (-)	101 (0.09)/0 (0)
*Sorex araneus*	0 (0)/0 (-)	79 (0.35)/0 (0)	222 (0.61)/0 (0)	33 (0.21)/0 (0)	10 (0.06)/0 (0)	344 (0.31)/0 (0)
*Cricetulus triton*	0 (0)/0 (-)	141 (0.62)/0 (0)	248 (0.69)/0 (0)	49 (0.31)/0 (0)	16 (0.10)/0 (0)	454 (0.41)/0 (0)
*Cricetulus arabensis*	4 (0.02)/0 (0)	35 (0.15)/0 (0)	4 (0.01)/0 (0)	3 (0.02)/0 (0)	23 (0.14)/0 (0)	69 (0.06)/0 (0)

a Zero divided by zero cannot be calculated, so the result is replaced by a symbol.
